# Expanded access to publicly subsidised lisdexamfetamine treatment for adults with attention-deficit/hyperactivity disorder: An interrupted time-series analysis

**DOI:** 10.1177/00048674231170556

**Published:** 2023-04-25

**Authors:** Claudia Bruno, Helga Zoega, Malcolm B Gillies, Alys Havard, David Coghill, Sallie-Anne Pearson, Jonathan Brett

**Affiliations:** 1 School of Population Health, Faculty of Medicine & Health, University of New South Wales, Sydney, NSW, Australia; 2 Centre of Public Health Sciences, Faculty of Medicine, University of Iceland, Reykjavík, Iceland; 3 National Drug and Alcohol Research Centre, Faculty of Medicine & Health, University of New South Wales, Sydney, NSW, Australia; 4 Departments of Paediatrics and Psychiatry, Faculty of Medicine, Dentistry and Health Sciences, The University of Melbourne, Melbourne, VIC, Australia; 5 Murdoch Children’s Research Institute, Melbourne, VIC, Australia

## Introduction

Attention-deficit/hyperactivity disorder (ADHD) and related impairments persist into adulthood for approximately two-thirds of people diagnosed in childhood (Di Lorenzo et al., 2021). ADHD medicine use among Australian adults doubled between 2013 and 2020 (Bruno et al., 2022); however, rates of publicly subsidised ADHD pharmacotherapy (0.3–1.0%) in 2020 have remained lower than the global prevalence of persistent adult ADHD from childhood (2.6%) (Song et al., 2021). While a proportion of adults may have been accessing privately funded pharmacotherapy not captured in prior Australian analyses, other barriers are likely contributing to this apparent treatment gap (Bisset et al., 2023).

Until recently, Australian adults with ADHD diagnosed after the age of 18 years were only eligible for subsidised access to immediate-release forms of dexamfetamine and methylphenidate. On the contrary, adults who were diagnosed in childhood could access all publicly subsidised ADHD medicines, including atomoxetine, guanfacine, lisdexamfetamine and long-acting methylphenidate. On 1 February 2021, Australia’s Pharmaceutical Benefits Scheme (PBS) listing for lisdexamfetamine was expanded to allow use in adults with ADHD persisting from childhood, even when diagnosed after 18 years of age. Lisdexamfetamine, a pro-drug of dexamfetamine, with a 12–13 hour duration of action is taken once daily, compared with immediate-release stimulants that require dosing two to three times per day.

Prior to these subsidy changes, an estimated 20,000 Australian adults were paying more than $1200 (AUD) per person each year for privately funded lisdexamfetamine treatment (Department of Health and Aged Care, 2021). Expansion of the PBS-listing for lisdexamfetamine was intended to increase treatment access for people who were diagnosed in adulthood; however, little is known about the actual impact on dispensing of lisdexamfetamine and other ADHD medicines.

Here, we provide contemporary prevalence and incidence estimates for ADHD medicine use among adults in Australia for 2021. We describe the changes in dispensing of subsidised lisdexamfetamine and other subsidised ADHD medicines for adults following the expanded PBS-listing of lisdexamfetamine.

## Methods

Services Australia maintains a dataset that includes all PBS dispensing claims for a randomly selected 10% sample of PBS-eligible Australians. We used the PBS 10% sample dataset to examine monthly dispensing rates of subsidised ADHD medicines between 1 February 2016 and 31 January 2022, among adults aged 18 and over. For the year 2021, we estimated the annual prevalence and incidence as described in our previous study (Bruno et al., 2022).

We used an interrupted time-series analysis to quantify changes in monthly dispensing following the lisdexamfetamine listing change on 1 February 2021. We calculated monthly dispensing rates by first multiplying the number of dispensings in each month by 10 before dividing by the estimated population of adults, interpolated from the Australian Bureau of Statistics quarterly population estimates (Australian Bureau of Statistics, 2022). We modelled the shape of the impact on dispensing following this event by including a term representing an immediate and sustained increase or decrease (step change) and a term representing a change in the trend. We used seasonal autoregressive integrative moving average (ARIMA) models of log-transformed dispensing rates to control for autocorrelation and seasonality (Schaffer et al., 2021).

We stratified analyses by ADHD medicine, age group and patient sex. We present all estimates per 1000 population and conducted our analyses using R version 4.2.1 (R Project for Statistical Computing). The code used in the analysis can be found at https://github.com/ClaudiaBruno/Lis_res_letter.

## Ethics statement

The New South Wales (NSW) Population and Health Services Research Ethics Committee approved this study (Approval Number: 2019/ETH01176) and the Australian Government Services Australia External Request Evaluation Committee granted data access (Approval Numbers: MI7542/RMS1941).

## Results

From 1 February 2016 to 31 January 2022, there were approximately 3,486,870 ADHD medicine dispensings to 199,280 adults (57% male). In 2021, the annual prevalence of ADHD medicine use was 6.8 per 1000 adults; it was higher among people aged 18–24 years (19.0 per 1000) than those ⩾ 25 years (5.5 per 1000). New use (incidence) was greater among females than males across both age groups, and lisdexamfetamine was the most initiated ADHD medicine followed by dexamfetamine (Table 1).

**Table 1.** Incidence and prevalence of ADHD medicine use per 1000 adults in Australia in 2021, by age, sex and ADHD medicine.

	Incidence (*n*)	Prevalence (*n*)
**Overall (⩾** **18** **years)**	**2.2 (44,740)**	**6.8 (136,770)**
**Young adults 18–24** **years**	**5.1 (11,300)**	**19.0 (41,950)**
Male	3.9 (4460)	20.4 (23,140)
Female	6.4 (6840)	17.6 (18,810)
Male to female sex ratio	0.6	1.2
**Adults** **⩾** **25** **years**	**1.9 (33,440)**	**5.5 (97,590)**
Male	1.8 (15,720)	6.1 (52,980)
Female	1.9 (17,720)	4.9 (44,610)
Male to female sex ratio	0.9	1.2
**Medicine initiated on**^a^/dispensed		
*Stimulants*	*3.3 (67,020)*	*7.7 (153,410)*
Dexamfetamine	1.0 (19,610)	3.0 (60,750)
Lisdexamfetamine	1.6 (33,050)	2.4 (47,800)
Methylphenidate^b^	0.7 (14,360)	2.2 (44,860)
*Non-stimulants*	*0.3 (5260)*	*0.5 (10,160)*
Atomoxetine	0.2 (3310)	0.3 (6350)
Guanfacine	0.1 (1950)	0.2 (3810)
**Total population**, **⩾** **18 years**^c^	**20,039,723**	

ADHD: attention-deficit/hyperactivity disorder. Bold or italic is to symbolise that they are the overall estimate of the below categories. For example - young adults 18–24 years - includes both male and females.

a People can initiate on more than one medicine per year, lookback is medicine-specific for each stratum.

b Methylphenidate represents both immediate-release and long-acting methylphenidate formulations. Long-acting methylphenidate currently listed only for people with ADHD who were diagnosed between 6 and 18 years (inclusive).

c Raw numbers are multiplied by 10 as our data are based on a 10% sample of Australians.

**Figure 1.**
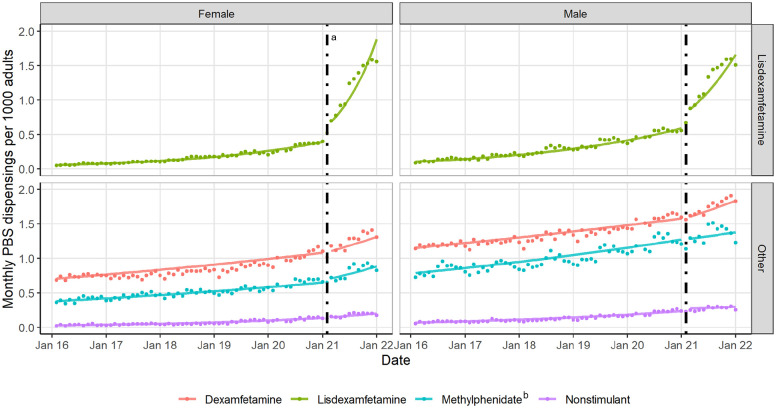
Monthly rate of ADHD medicine dispensings per 1000 Australian adults, observed (dotted) and predicted (solid line) trends from 1 February 2016 to 31 January 2022. ADHD, attention-deficit/hyperactivity disorder. ^a^Dashed line – 1 February 2021 represents the date the subsidy listing for lisdexamfetamine was expanded to include adults who were diagnosed with ADHD after age 18 years. ^b^Methylphenidate represents both immediate-release and long-acting methylphenidate formulations. Long-acting methylphenidate is currently listed only for people with ADHD who were diagnosed between 6 and 18 years (inclusive).

We found the public subsidy change was associated with an immediate and sustained increase in monthly lisdexamfetamine dispensings of 37% (95% confidence interval [CI]: [23%, 53%]), with an increase in trend through to the end of the study period of an additional 5% per month (95% CI: [3%, 7%]) on top of pre-existing monthly increases of 3.14% (95% CI: [3.04%– 3.23%]). Specifically, the mean monthly dispensing rate for lisdexamfetamine increased to 1.2 per 1000 adults in the 12 months following the intervention compared to 0.4 per 1000 in the 12 months prior. We did not observe either step or trend changes in other stimulant (dexamfetamine and methylphenidate) or non-stimulant medicine dispensing. While immediate and sustained increases in lisdexamfetamine dispensing were similar for females and males (43%, 95% CI: [26%, 63%] vs 29% [18%, 41%]), females had higher trend increases through to the end of the study period of 7% per month (95% CI: [5%, 9%]) compared to males 4% [2%, 5%] (Figure 1).

## Discussion

It is clear from our analysis that expanding subsidy listing of lisdexamfetamine resulted in increased dispensing of subsidised lisdexamfetamine among adults with ADHD. Furthermore, lisdexamfetamine dispensing continued to rise each month throughout the following year at an increased rate. Less clear are the underlying prescribing phenomena driving these changes. The immediate and sustained increase in dispensing is unlikely to be related to an immediate increase in new people treated. More likely, this is due to people switching from unrecorded privately prescribed lisdexamfetamine to the public market. This could be confirmed using medicines sales data or State and Territory stimulant prescription monitoring data.

Continued monthly increases in lisdexamfetamine following this initial jump in dispensings may represent increasing awareness of ADHD persisting into adulthood among psychiatrists, general practitioners, and the public leading to improved recognition and diagnosis. The use of lisdexamfetamine for all ages was already increasing before the subsidy listing was expanded (Bruno et al., 2022), and this growing market share may indicate a prescriber or patient preference for lisdexamfetamine. Recently updated Australian guidelines for ADHD management (Australian ADHD Guideline Development Group, 2022) do not recommend a particular stimulant as first-line treatment in adults. Rather, they acknowledge the benefits and downsides of each stimulant medicine and formulation, noting clinical decisions should also consider the needs of the person with ADHD. However, lisdexamfetamine is the only psychostimulant still on patent and therefore actively promoted and marketed by the pharmaceutical sponsor; promotion about convenient dosing, improved treatment adherence and lower abuse potential may contribute to increases in market share.

Notwithstanding increases in lisdexamfetamine, the rates of ADHD medicine use in adults in 2021 remain below the estimated prevalence of ADHD (Song et al., 2021). Following the expansion of subsidy for lisdexamfetamine, the Pharmaceutical Benefits Advisory Committee have recommended long-acting methylphenidate also be subsidised for adults diagnosed with ADHD after the age of 18 years (Pharmaceutical Benefits Advisory Committee [PBAC], 2022). Several other barriers to treatment access for adults with ADHD exist in Australia. ADHD diagnosis and psychostimulant prescribing are generally restricted to paediatricians and psychiatrists most of whom work in private practice, have long waiting lists and charge substantial out-of-pocket fees. Hence, access to specialist care disproportionately impacts people of lower socioeconomic status and those in regional and remote areas of Australia, further contributing to existing inequities. We were unable to account for these factors using the PBS 10% dataset. Nevertheless, the subsidy change described here is a step in the right direction to improving access to treatment for adults with ADHD persisting from childhood.
